# Combating hepatitis B and C by 2030: achievements, gaps, and options for actions in China

**DOI:** 10.1136/bmjgh-2020-002306

**Published:** 2020-06-30

**Authors:** Shu Chen, Wenhui Mao, Lei Guo, Jiahui Zhang, Shenglan Tang

**Affiliations:** 1 Global Health Research Center, Duke Kunshan University, Kunshan, Jiangsu, China; 2 Duke Global Health Institute, Duke University, Durham, North Carolina, USA; 3 Research Department of Social Development, Development and Research Center of State Council, Beijing, China

**Keywords:** viral hepatitis, health systems

## Abstract

China has the highest number of hepatitis B and C cases globally. Despite remarkable achievements, China faces daunting challenges in achieving international targets for hepatitis elimination. As part of a large-scale project assessing China’s progress in achieving health-related Sustainable Development Goals using quantitative, qualitative data and mathematical modelling, this paper summarises the achievements, gaps and challenges, and proposes options for actions for hepatitis B and C control. China has made substantial progress in controlling chronic viral hepatitis. The four most successful strategies have been: (1) hepatitis B virus childhood immunisation; (2) prevention of mother-to-child transmission; (3) full coverage of nucleic acid amplification testing in blood stations and (4) effective financing strategies to support treatment. However, the total number of deaths due to hepatitis B and C is estimated to increase from 434 724 in 2017 to 527 829 in 2030 if there is no implementation of tailored interventions. Many health system barriers, including a fragmented governance system, insufficient funding, inadequate service coverage, unstandardised treatment and flawed information systems, have compromised the effective control of hepatitis B and C in China. We suggest five strategic priority actions to help eliminate hepatitis B and C in China: (1) restructure the viral hepatitis control governance system; (2) optimise health resource allocation and improve funding efficiency; (3) improve access to and the quality of the health benefits package, especially for high-risk groups; (4) strengthen information systems to obtain high-quality hepatitis epidemiological data; (5) increase investment in viral hepatitis research and development.

**Summary box**

- China has made considerable achievements in controlling hepatitis B and C through multiple strategies with efforts focused on prevention and increased treatment financing.
- Formidable challenges remain in combating hepatitis by 2030. Key health system barriers, including a fragmented governance system, insufficient funding, inadequate service coverage and unstandardised treatment, and flawed information systems, have compromised the effective control of viral hepatitis.
- To tackle these challenges, China must take five immediate actions: restructuring the governance system of viral hepatitis, optimising resource allocation and increasing the efficiency of funding, improving access to and the quality of the health benefits package, strengthening information systems and boosting investment on hepatitis research and development.

## Introduction

Infection with chronic viral hepatitis can be caused by exposure to five different types of viruses (hepatitis A, B, C, D, E). Hepatitis B virus (HBV) and hepatitis C virus (HCV) account for 96% of all deaths related to viral hepatitis.^1^ China is the country experiencing the highest burden of these infections,^2 3^ with the WHO estimating that in 2016, 90 million people were living with chronic HBV infection and 10 million with chronic HCV infection in China, accounting for one-third and 7% of the global infections, respectively.^4^ Chronic HBV and HCV infection can progress to cirrhosis, hepatocellular carcinoma and premature death without proper treatment.^5^ Chronic HBV infections are associated with increased risk of other cancers including stomach cancer, colorectal cancer, oral cancer, pancreatic cancer and lymphoma.^6^ Among people living with chronic HBV and HCV, around 7 million and 2.5 million needed urgent treatment in China due to advanced liver diseases or the high risk of developing into cancer, respectively, in 2016.^4^ In 2017, there were an estimated 310 079 and 124 645 deaths due to chronic HBV and HCV infections, respectively, in China, according to the Global Burden of Diseases (GBD) 2017 Study.^7^

Viral hepatitis control in China is governed by the Bureau of Disease Prevention and Control, National Health Commission (NHC) and overseen by health commissions at the provincial, prefecture and county levels across the country. Under the regulatory supervision of NHC, the Chinese Center for Disease Control and Prevention (China CDC) is responsible for disease prevention and management, while hospitals provide clinical diagnosis and treatment. The Division of Immunization Planning Management and Division of HIV/AIDS Prevention and Control within NHC is responsible for hepatitis B and C control, respectively. The same governance structure for hepatitis B and C control has been put in place at the China CDC system nationwide.

Viral hepatitis is increasingly garnering global attention and is included in the United Nations’ 2030 Agenda for Sustainable Development Goals (SDGs) where SDG 3.3 calls for ‘combat viral hepatitis’.^8^ At the same time, in 2016, WHO published its first Global Health Sector Strategy on Viral Hepatitis 2016–2021, which established nine quantitative global targets, such as ‘reducing new cases of chronic viral hepatitis B and C infections by 90% and deaths by 65% by 2030’.^9^ The first Action Plan for the Prevention and Treatment of Viral Hepatitis in China (2017–2020) was jointly published by 11 ministries in 2017, which set out 6 targets, 4 of which corresponded with WHO’s targets (table 1).^10^ Despite the priorities and action recommendations put forward by the international community to eliminate hepatitis globally and analysis of eliminating hepatitis B in China,^11 12^ we present key achievements, identify gaps and challenges, and proposes next steps to specifically help China end hepatitis B and C as a major public health threat by 2030.

**Table 1:** The hepatitis targets set by WHO and China

Target area	WHO 2020 targets (base year: 2015)	WHO 2030 targets (base year: 2015)	China 2020 targets
Impact targets			
Incidence: new cases of chronic viral hepatitis B and C infections	30% reduction (equivalent to 1% prevalence of HBsAg among children)	90% reduction (equivalent to 0.1% prevalence of HBsAg among children)	Keeping <1% prevalence of HBsAg among children under 5
Mortality: viral hepatitis B and C deaths	10% reduction	65% reduction	No quantitative target
Service coverage targets			
HBV vaccination: childhood third dose vaccination coverage	90%	90%	Keeping >95%
Prevention of HBV mother-to-child transmission: HBV birth-dose vaccination coverage or other approaches to prevent mother-to-child transmission	50%	90%	Keeping >90%
Blood safety	95% of donations screened in a quality-assured manner	100% of donations screened in a quality-assured manner	100% of donations screened in a quality-assured manner
Safe injections: percentage of injections administered with safety-engineered devices in and out of health facilities	50%	90%	No quantitative target
Harm reduction: number of sterile needles and syringes provided per person who injects drugs per year	200	300	No quantitative target
Viral hepatitis B and C diagnosis	30%	90%	No quantitative target
Viral hepatitis B and C treatment	Globally 5 million people receiving HBV treatment and 3 million people receiving HCV treatment	80%	No quantitative target
China-specific service coverage targets			
Public awareness of viral hepatitis prevention and control knowledge			>50%
Drug dependence treatment coverage to opioid users			>70%

Sources: Global health sector strategy on viral hepatitis 2016–2021: towards ending viral hepatitis & Action plan for the prevention and treatment of viral hepatitis in China (2017–2020).

HBsAg, HBV surface antigen; HBV, Hepatitis B virus; HCV, hepatitis C virus.

## Approach

This article collected quantitative and qualitative data for analysis. Quantitative data were collected from published literature in Chinese and English, GBD 2017 Study estimates,^3^ the infectious diseases surveillance reporting system (IDSRS) and reports published by related governmental agencies. The health outcome projection results are estimated using the adjusted model developed by the GBD SDG team.^13^ Also, this paper includes qualitative findings from nine interviews purposively conducted among key stakeholders, including policy-makers, hepatitis control professionals and clinicians at national and provincial levels from Jiangsu, Hubei and Yunnan provinces representing eastern, central and western China, respectively, in 2017. The current study is part of a large-scale project assessing the progress of China in achieving health-related SDGs, and which has published detailed methods on the qualitative data collection and analysis and the projection model.^14^

## Achievements in hepatitis B and C control in China

China has made substantial progress in controlling HBV and HCV infections over the past few decades. Based on the national seroepidemiology surveys conducted by China CDC since the 1990s, the seroprevalence of HBV surface antigen (HBsAg) among children <15 years old has declined from 10.5% in 1992 to 0.8% in 2014, and reached 0.3% among children under age 5 in 2014.^15^ The overall seroprevalence of anti-HCV antibody fell from 3.2% in 1996 to 0.43% in 2006.^16 17^ These achievements in China may, to a large extent, be attributed to the four national programmes or policies as outlined below.

First, China’s HBV childhood immunisation programme has been recognised by WHO for its remarkable success.^18^ China was among the first developing countries to establish an HBV immunisation programme in 1992, recommending timing the first dose vaccination within 24 hours of birth and the second and third doses at 1 month and 6 months of birth, respectively.^19^ In 2002, with financial support from both the GAVI Alliance and the Chinese Government, the HBV vaccine was included in the National Immunization Program and made available free of charge to all newborns by 2005.^20^ An estimated total of ¥20.3 billion (¥1≈ US$0.14) was allocated by the central government to support the programme between 1992 and 2005.^21^ In 2009, the programme was further expanded to vaccinate children aged ≤15 years, with 68 million children successfully vaccinated.^22^ Thanks to the effective implementation of this policy, the three-dose vaccine coverage tripled from 30.0% in 1992 to 99.6% in 2015 with the timely first dose rate increasing from 22.2% to 95.6% during the same period.^23^

Second, comprehensive programmes to prevent mother-to-child transmission boosted HBV transmission control. Mother-to-child transmission is estimated to be responsible for 40%–50% of new HBV infections in China.^24^ Beginning in 2011, China initiated a national programme for integrated prevention of mother-to-child transmission (PMTCT) of HIV, syphilis and HBV. The programme includes free HBV screening services during pregnancy and administration of hepatitis B immunoglobulin within 12 hours of birth for babies born to HBV-infected mothers. It was expanded nationwide in 2015, and around ¥1.4 billion has been invested annually to cover the HBV-related services, which reached 95.6% coverage in 2015.^25 26^

Third, full coverage of nucleic acid amplification testing (NAT) in all blood stations substantially improved blood safety and prevented transfusion-transmitted infections, including HBV and HCV. The probability of transmitting via blood is especially high for HCV, and NAT can detect low levels of virus during a window period. NAT was included in routine donor screenings and piloted in 14 selected blood stations of 11 provinces since 2010.^27^ This practice was expanded nationwide in 2014 and received 1 billion RMB from the central budget to fund implementation in all blood stations.^28^

Lastly, China has made remarkable progress in implementing effective financing policies to make HBV and HCV drugs affordable over the past few years. Entecavir and tenofovir, two WHO-recommended first-line HBV drugs, were included in the updated National List of Reimbursable Medicines (NLRM) in February 2017.^29^ The two drugs were further selected into the ‘4+7 Cities Centralized Drug Procurement Document’, published in November 2018, a novel procurement pilot scheme aiming to dramatically cut the price paid for generic drugs through centralised joint procurement.^30^ Due to these efforts, the prices of entecavir and tenofovir have been substantially reduced, especially in the ‘4+7 Cities’ where it only takes around ¥18 (less than US$3) to complete 1 month entecavir or tenofovir treatment. By 2017, all the antiviral drugs recommended by the Chinese guidelines to treat HBV, including interferon-alfa, pegylated interferon-alfa and five nucleoside analogues, had been included in the NLRM.^28^ Remarkably, in recent years, treatment for HCV has vastly improved, and the sustained virological response rate of pan-genotypic Direct-Acting Antivirals (DAAs) has reached over 95%.^31^ Elbasvir–grazoprevir, ledipasvir–sofosbuvir and sofosbuvir–velpatasvir tablets can cure two, four and six main genotypes of HCV, respectively, though these medications are very expensive. They received expedited approval from the National Medical Products Administration to be marketed in China in 2018.^32^ The recent NLRM updated on 28 November 2019 has included the three HCV drugs, and the prices are expected to drop by 85% on average.^33 34^

## Gaps and challenges

**Figure 1:**
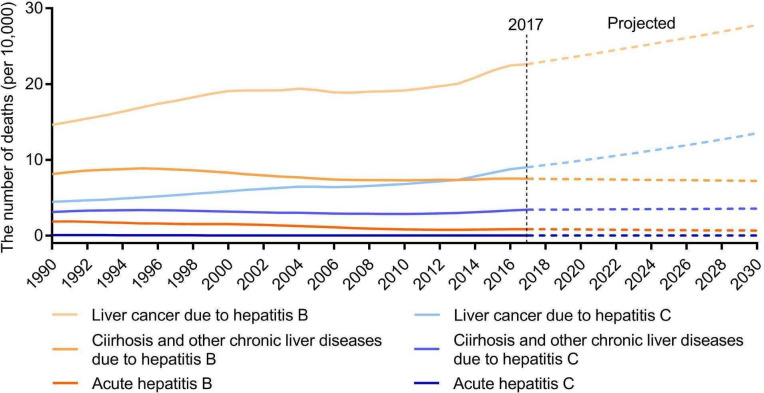
The historical change of deaths due to hepatitis B and C from 1990 to 2017 and projected changes from 2017 to 2030 in China. Data source: GBD 2017 Study.

China has almost reached WHO’s targets for HBsAg prevalence (0.3% in 2014 among children under 5), HBV vaccination (99.6% in 2015), PMTCT (95.6% in 2015) and blood safety (100% blood screening since 2014; See table 1 for specific target definition).^15 23 28^ However, progress has been much slower in reaching the remaining five targets surrounding deaths, diagnosis and treatment of hepatitis B and C, and safe injections and harm reduction. China’s Hepatitis Action Plan has not established any quantitative goals to close this gap. Based on data available, we were only able to project the number of expected deaths. It is estimated that the total number of deaths due to hepatitis B and C would increase from 434 724 in 2017 to 527 829 in 2030. Particularly, mortality will be predominately driven by liver cancer caused by hepatitis B and C (figure 1). Based on our interviews, reversing this trend cannot be done without addressing the following challenges.

The governance system of viral hepatitis control is fragmented in China. There is no specific single department or unit within the NHC or CDC to plan and lead hepatitis control work centrally. Currently, viral hepatitis is managed by different departments at the NHC or CDC based on similar transmission patterns or control strategies with other diseases. The Division of Immunization Planning Management leads HBV prevention through the vaccination programme, while the Division of HIV/AIDS Prevention and Control leads hepatitis C prevention given similar transmission patterns between HIV and HCV. The fragmented governance system has resulted in a lack of central strategic planning and leadership, which has further led to inadequate financial and personnel support. For example, we found through our interviews that hepatitis C control is highly dependent on the work plan, budget, skills and personnel of HIV/AIDS.

Health financing is insufficient to fund the elimination of hepatitis B and C in China. Prevention and control strategies rely on domestic governmental funding that currently covers vaccination and PMTCT services of hepatitis B. However, a minimal amount of funding is explicitly allocated to hepatitis C, except for that bundled within HIV/AIDS control. Consequently, there is a massive gap in funding for crucial routine work such as disease surveillance (eg, the HBV seroepidemiology surveys have been funded by the Ministry of Science and Technology through research project applications), and the gap is even more massive for hepatitis C control with little support to implement effective prevention strategies. Although recommended HBV drugs have been included in the NLRM for over 3 years, the actual reimbursement rates vary greatly across China due to the decentralised management and risk pooling across Chinese health insurance schemes. This policy poses formidable challenges in achieving universal hepatitis treatment. It will be the same case for the HCV drugs recently added into the NLRM if no effective actions are taken.

The delivery of comprehensive health services for hepatitis B and C, including prevention, testing, diagnosis and treatment, has not achieved its ideal coverage and standardisation. Hospital-acquired infections have not been prevented in low-tiered hospitals or hospitals in remote areas, which may follow less strict sterilisation procedures and skip testing HCV before surgical operations. The coverage of harm reduction practices to prevent HCV infection among drug users is low given stigma, discrimination and legality of using drugs.^11^ HBV testing was removed from routine health check-ups for new employees and students in 2010 to avoid discrimination against people infected with HBV.^35^ The diagnosis and treatment rate of hepatitis B and C remains extremely low according to our interviews, though there is no public data available to confirm this. For people who are diagnosed and treated, overtreatment is common with hospitals in China incentivised to prescribe drugs, particularly auxiliary medications to generate income.

The information system of viral hepatitis is unable to provide adequate data on the hepatitis epidemic for strategic planning. The current information system, IDSRS, primarily captures HBV and HCV new infections and deaths while providing little information on critical indicators such as service coverage, diagnosis and treatment. Duplication of data undermines its quality and accuracy.

## Options for actions towards eliminating viral hepatitis B and C in China

In light of the above challenges and taking into account recommendations from literature and key stakeholder interviews, we propose five strategic priority actions to help achieve the international and domestic targets and eliminate viral hepatitis in China.

First, restructure the governance system for viral hepatitis control within NHC and the China CDC to change the current fragmented management situation. It is suggested that the NHC establish a separate and independent division for viral hepatitis under the Disease Control Bureau to lead the national viral hepatitis control effort, with the Health Commission and CDC at different administrative levels making similar structural changes. The department should have its own team, budget and work plan with performance indicators to ensure successful operation.

Second, optimise resource allocation and increase the efficiency of funding to ensure sufficient and sustainable health financing for hepatitis elimination. It is essential that the NHC identify the funding required to achieve targets, and develop a comprehensive and feasible hepatitis control budget to cover the continuum of health services. The NHC worked with WHO to develop an investment plan for hepatitis in 2016, though it remains unclear if and how this plan will be used for strategic planning. Meanwhile, the NHC can work with the Ministry of Finance and the National Healthcare Security Administration on funding resource mobilisation and reallocation for sustained viral hepatitis health financing, ideally under the designated leadership of the State Council. It is essential to standardise treatment and reduce drug prices to increase funding efficiency. Regional disparities in health insurance coverage need to be assessed and addressed through central budgeting compensations.

Third, improve access to and quality of the health benefits package for patients with viral hepatitis, especially for high-risk groups. The five core interventions proposed by WHO (box 1) to be included in benefits packages are available in China, while extra efforts need to be invested in strengthening surgical safety and other sources of hospital-acquired infections and harm reduction services.^9^ It is critical to increase the identification of the number of infected individuals with an effective disease surveillance system using better testing and diagnosis accuracy, coverage and reporting. It is vital to increase the treatment rate by developing a standard treatment package with sufficient financial coverage, particularly for low-income populations, and an individualised patient management system, especially for HBV lifelong treatment (box 2). High-risk populations, including healthcare workers, people born to mothers with hepatitis B, injection drug users, indigenous peoples and ethnic minorities, prisoners, migrants, men who have sex with men, persons coinfected with HIV and hepatitis, and blood donors need to be identified and prioritised for specific prevention, testing, diagnosis, care and treatment.^9^ Tailored interventions for these high-risk populations can be implemented to microeliminate hepatitis within the discrete group.^36^ It is important to address HBV and HCV-related stigma and discrimination, through identifying the drivers of this discrimination, alongside population-wide health education programmes.

Box 1 **Five core viral hepatitis interventions proposed by WHO**

- HBV vaccination;
- Injection, blood and surgical safety and universal precautions;
- Prevention of mother-to-child transmission of hepatitis B virus;
- Harm reduction services for people who inject drugs;
- Treatment of chronic hepatitis B virus and hepatitis C virus infection.

Source: Global Health Sector Strategy on Viral Hepatitis, 2016–2021, WHO

Box 2 **Hepatitis B and C treatment challenges and proposed options for actions in China**

Effective prevention interventions have reduced new hepatitis B virus (HBV) infections and put new hepatitis C virus (HCV) infections under control in China. Treating the large number of people with HBV and HCV needs to be prioritised to meet viral hepatitis elimination targets.

Addressing low hepatitis B and C treatment rates largely due to inadequate case identification and high patient financial burden face formidable challenges with respect to service coverage, service quality and financial protection. The treatment success rate is an issue for people infected with hepatitis B, who have to receive lifelong treatment despite the asymptotic nature and side effects. The quality of service provided varies by hospital, and there are no standardised treatment packages. Overprescription of medicines is prevalent, especially auxiliary medicines, as a way to generate revenues. Effective financing strategies have improved the financial protections for patients with hepatitis, especially for patients with hepatitis B in the ‘4+7 Cities’.

Looking ahead, China must take urgent actions to improve the treatment service coverage, quality and affordability for people infected with HBV and HCV. Key recommendations are:

- Increase identification of infected individuals through a better surveillance system and improved testing and diagnosis accuracy. Resource-rich provinces and cities can implement active patient identification strategies, while working on removing stigma and discrimination through population-wide health education programs.
- Develop a standardised treatment package and ensure it is well implemented at different levels of clinical institutions. Considering the root cause of overprescription, strategies to rationalise physicians’ salary and apply the diagnosis-related groups or capitation payment need to be in place. Capacity training for physicians and carefully designed performance evaluation indicators are also essential to ensure the successful implementation.
- Strengthen patient management to improve medication adherence. This is especially important for patients with hepatitis B. Hospitals should apply individual patient management to them, similar to the treatment and management of patients with HIV/AIDs.
- Decrease patient financial burden due to treatment, especially for low-income groups. Economic analysis has demonstrated high cost effectiveness of investing HBV and HCV treatment drugs in many countries. Despite the huge progress, continuing investment needs to be made on reducing drug price and increasing reimbursement rate especially for the low-income patients to increase equity.

Fourth, strengthen information systems to obtain high-quality hepatitis epidemiological data. A well-designed quality control system needs to be established and implemented to avoid inaccurate and duplicate cases of hepatitis infection and hepatitis-related death. The system should also integrate process indicators for service coverage to capture data throughout the continuum of care, making it possible to assess the national hepatitis burden and monitor access to, uptake of and quality of services delivered.

Lastly, boost investment for hepatitis research and development (R&D) innovations. It is unlikely for either the world or China to eliminate the viral hepatitis burden unless significant progress is made to develop new medicines, technologies and innovative service delivery approaches. Continuing and increasing investment is necessary to improve the prevention, testing, diagnostics, treatment and patient management of chronic viral hepatitis. Particularly, priority should be placed on R&D in effective HCV vaccination and short-course HBV curative treatments.

## Conclusion

This article highlights recent improvements in the control of hepatitis B and C in China and how they can be furthered. Given projections for increased deaths due to hepatitis B and C in China and the large numbers of individuals infected with hepatitis B and C, it is critical to urgently develop and implement a set of concerted actions, as proposed above, with adequate resources put in place to support the effective implementation of these actions. Inaction, or delay in taking these actions, would have to make a negative impact on a large number of Chinese households and society.
